# Peptidomic and glycomic profiling of commercial dairy products: identification, quantification and potential bioactivities

**DOI:** 10.1038/s41538-019-0037-9

**Published:** 2019-03-14

**Authors:** Mrittika Bhattacharya, Jaime Salcedo, Randall C. Robinson, Bethany Michele Henrick, Daniela Barile

**Affiliations:** 1 Department of Food Science and Technology, University of California Davis, Davis, CA 95616 USA; 2 Evolve Biosystems, 2121 2nd Street, B107, Davis, CA 95618 USA; 3 Department of Food Science and Technology, University of Nebraska Lincoln, Lincoln, NE 68588 USA

**Keywords:** Peptides, Mass spectrometry, Glycomics

## Abstract

Peptidomics and glycomics are recently established disciplines enabling researchers to characterize functional characteristics of foods at a molecular level. Milk-derived bioactive peptides and oligosaccharides have garnered both scientific and commercial interest because they possess unique functional properties, such as anti-hypertensive, immunomodulatory and prebiotic activities; therefore, the objective of this work was to employ peptidomic and glycomic tools to identify and measure relative and absolute quantities of peptides and oligosaccharides in widely consumed dairy products. Specifically, we identified up to 2117 unique peptides in 10 commercial dairy products, which together represent the most comprehensive peptidomic profiling of dairy milk in the literature to date. The quantity of peptides, measured by ion-exchange chromatography, varied between 60 and 130 mg/L among the same set of dairy products, which the majority originated from caseins, and the remaining from whey proteins. A recently published bioactive peptide database was used to identify 66 unique bioactive peptides in the dataset. In addition, 24 unique oligosaccharide compositions were identified in all the samples by nano LC Chip QTOF. Neutral oligosaccharides were the most abundant class in all samples (66–91.3%), followed by acidic (8.6–33.7%), and fucosylated oligosaccharides (0–4.6%). Variation of total oligosaccharide concentration ranged from a high of 65.78 to a low of 24.82 mg/L. Importantly, characterizing bioactive peptides and oligosaccharides in a wider number of dairy products may lead to innovations that go beyond the traditional vision of dairy components used for nutritional purposes but that will rather focus on improving human health.

## Introduction

Milk is an essential food fulfilling the nutritional requirement of the neonate, and its composition has been shaped to promote species’ survival as a result of 200 million years of evolution. Milk is a source of macronutrients including lactose, lipids and proteins, but also contains vitamins, minerals, oligosaccharides (OSs), innate immune factors, immunoglobulins, hormones, enzymes and growth factors critical to neonatal health.^1^ These components play a pivotal role in various functions of the body such as cardiovascular, immunomodulation, metabolic, and neuronal development, as well as establishing the gut microbiome.^2–5^

Milk proteins are a potential source of bioactive peptides. These bioactive peptides are short amino acidic sequences encrypted in milk proteins and can be released in vivo during gastrointestinal digestion of milk, or in vitro by fermenting milk with proteolytic starter cultures (lactic acid bacteria**)**, or by enzymatic hydrolysis.^6,7^ The released bioactive peptides are mostly short in size, ranging from 2 to 50 amino-acid residues.^8^ Many of these peptides, isolated from bovine milk or synthesized de novo, have been proven to affect the cardiovascular, neural, digestive, endocrine and immune systems in our body by exerting functional properties such as anti-hypertensive, anti-microbial, anti-oxidant, anti-thrombotic, immunomodulatory and opioid activities.^5^ Due to an increased prevalence of hypertension worldwide, researchers have primarily focused on the study of anti-hypertensive peptides and their role in cardiovascular diseases.^6^ The potential impact of these anti-hypertensive peptides in therapeutic applications has been well established.^9,10^ Furthermore, anti-hypertensive activity is one of many peptide functionalities, and it is possible that additional, equally valuable applications of dairy-derived peptides will be identified as the less-studied bioactivities are explored in greater depth.

OSs are the third most abundant component in human milk and are known to exert several beneficial effects such as providing direct immunomodulation of the intestinal mucosa, acting as decoy receptors against potentially pathogenic bacteria, promotion of brain development, and may be most importantly providing a critically important carbon source (prebiotic) for the establishment of the gut microbiome.^3,11^ They are composed of a lactose core decorated with several building block monosaccharides (hexose or Hex (galactose, glucose), HexNAc (*N*-acetylglucosamine), fucose, and sialic acid), which are joined by a variety of linkages, yielding highly complex and diverse structures. OSs are classified based on the composing monosaccharides: there are neutral OS (containing only glucose/galactose/*N*-acetylglucosamine), neutral fucosylated OS (similar to neutral OS but further decorated by fucose) and acidic/acidic fucosylated (OS based on a neutral core structure that also contains sialic acid and/or fucose). Their concentration varies greatly depending on the species, with human milk containing the highest amount (5–20 g/L). Human milk OSs are predominantly of the neutral fucosylated type (up to 60–70%) with acidic OS representing the remaining portion.^12^ In other mammalian species, OS concentration is much lower: bovine colostrum contains 1 g/L and their concentration decreases during lactation, reaching values lower than 100 mg/L^13^ in mature bovine milk. Regarding the diversity of structures, human milk still represents the gold standard, with over 150 structures identified; it is followed by bovine milk, with 55 unique structures described,^14^ and porcine milk, where 39 OSs were identified.^14,15^

In recent years, China’s dairy market has grown substantially and is expected to continue to expand. The USDA Global Agricultural Information Network report predicts an increase in fluid milk consumption in China to 38 million tons in the year 2018, which represents a major growth compared with a historically low base, as consumers are acquiring a new taste for dairy. Fluid shelf-stable milk and yogurt accounts for 65% of the market share in China.^16^ Further, it is predicted that over the next 3 years, China’s share of the world dairy market will equal that of the United States (14% of the world dairy market).^17^ Of note, China is the most dynamic segment of the global dairy market with intense product development, which led to the diversification of specialized dairy formulations targeted to specific age groups to meet their unique nutritional needs. Concurrently, a growing interest among consumers for solving health issues by making conscious eating decisions may lead them to prefer food containing naturally derived peptides and naturally existing OSs over synthetically derived pharmaceuticals, especially if comparable efficacy was established for targeted applications.

Hence, understanding the baseline presence of bioactive peptides and OSs could reveal opportunities for tailored processing to further increase peptide and/or OSs content and develop enriched milk-based products with health-promoting activities. The objective of this study was to profile the peptide and OS content of commercial dairy products in the Chinese marketplace and identify their functionalities. The methodologies employed for the research have the potential to improve our understanding of the bioactive content of dairy products and could guide the development of novel functional products in the future.

## Results

The results of the present study are divided in two parts: peptidomic and glycomic (OS) profiling. The main characteristics of 10 commercial dairy products are shown in Table 1.

**Table 1:** Commercial dairy products used in this study, grouped by target consumers and customization

Sample	Target consumer	Customization
Deluxe Organic Milk	Elderly	Organic pasture
Huanqing for bone	Elderly	Vitamin D, casein phosphopeptides, inulin
Pure Milk	All age groups	No supplementation
Original Yoghurt	All age groups	Raw milk fermented by lactic acid bacteria prior to sterilization
Xin Yang Dao	Lactose intolerant	Lactose hydrolysis
Chunzhen (Yoghurt)	25–42 years	Pasture milk, bacterial culture
Future Star for brain	Kids (3–12)	Pasture milk, DHA (docosahexaenoic acid) from algae oil, taurine
Future Star for bone	Kids (3–12)	Pasture milk, DHA (docosahexaenoic acid) from algae oil, vitamin A, vitamin D
Deluxe Milk	All age groups	High calcium
Huanqing for heart	Elderly	Vitamin E, fish oil extract

Peptidomic and glycomic profiling of these commercial dairy products were performed using state of the art mass spectrometry, ion-exchange chromatographic techniques and bioinformatic tools

### Peptidomic profiling

Relative and absolute quantification of peptides was carried on 10 dairy products, which comprised 8 milk-based products and 2 yogurt samples (see Table 1 for product description and customization aspect).

### Identification and relative quantification of peptides by liquid chromatographic (LC)–Orbitrap tandem mass spectrometry (MS/MS)

**Fig. 1:**
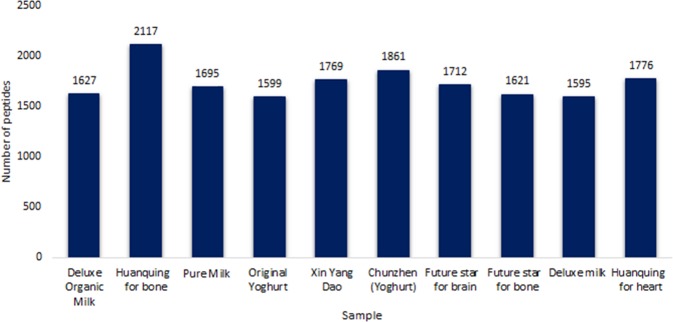
Total number of peptides identified by LC–Orbitrap MS/MS in the 10 commercial dairy products. The number of identified peptides, between 1595 and 2117 in the commercial dairy samples, represents a comprehensive peptidomic profiling of milk by employing high-resolution Orbitrap mass spectrometry

**Fig. 2:**
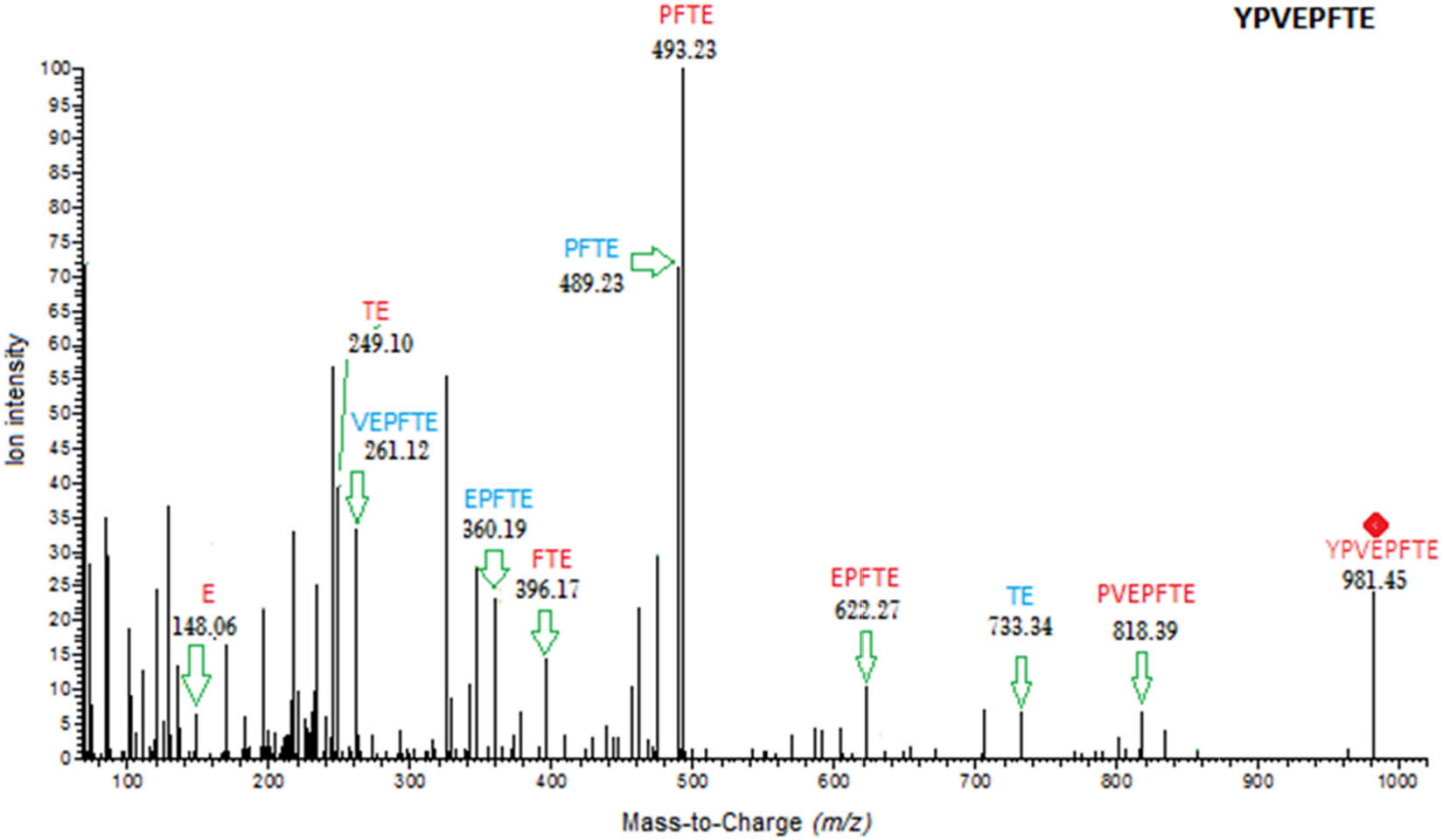
Tandem fragmentation of the anti-hypertensive peptide β-casein (129–136), m/z (*z* = 1) 981.45 at 35.48-min retention time with y-type ions in red, b-type ions in blue as identified by LC–Orbitrap MS/MS. The precursor ion, 981.45, is denoted by a red diamond

Peptide sequence length ranged from 6 to 50 amino acids. Over 1500 peptides were identified in each commercial dairy product (Fig. 1). The masses of the identified peptides ranged from 550.29 to 6008.15 Da. The highest and lowest number of peptide sequences were identified in the products named *Huanqing for bone* (2117 peptides) and *Deluxe Milk* (1595 peptides), respectively. As an example of the peptide identification process, Fig. 2 depicts an annotated tandem mass spectrometry (MS/MS) spectrum of β-casein (129–136), an anti-hypertensive peptide that has been correlated with improved cardiovascular health.^18^ Peptide fragments originating from the N-terminal and C-terminal are denoted as b-type and y-type ions, respectively. The identified peptide sequences for all samples are made available in Supplementary Table s1.

### Absolute quantification of peptides by ion-exchange chromatography

**Fig. 3:**
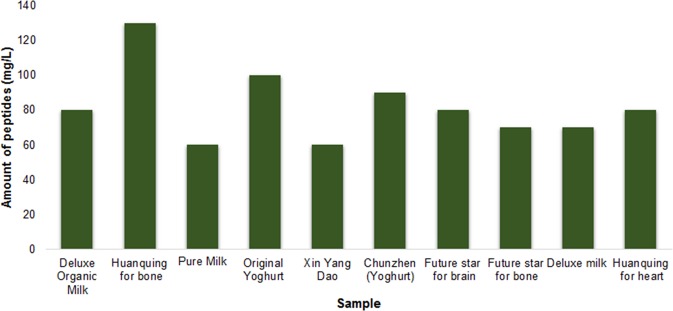
Amount of peptides (mg/L) as quantified by ion-exchange chromatography in the 10 commercial dairy products. The amount of peptides varied between 60 and 130 mg/L in same set of samples

The amount of the amino acids, representing peptide content varied greatly among the 10 commercial dairy products analyzed, ranging from 60 to 130 mg/L as measured by ion-exchange chromatography (Fig. 3). The lowest peptide content was observed in the sample *Pure Milk* (60 mg/L) and *Xin Yang Dao* (60 mg/L). *Huanqing for bone* had the highest concentration of peptides (130 mg/L). The absolute quantification results (mg/L) corroborated the relative quantification findings, which also identified the highest number of peptide sequences in the product named *Huanqing for bone*.

### Peptides derived from major and minor proteins

**Fig. 4:**
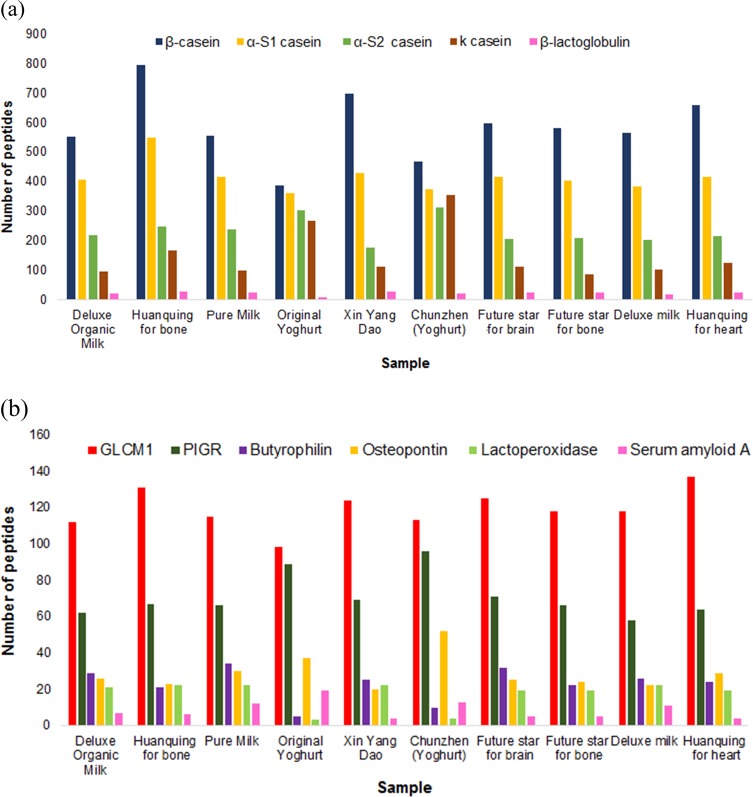
Major **a** and minor **b** proteins contributing to peptides formation as identified by LC–Orbitrap MS/MS in the 10 commercial dairy products. The majority of peptides derived from caseins, whereas whey proteins only minimally contributed to peptide formation

Our results indicate that the majority of peptides were derived from caseins. Whey proteins only minimally contributed to peptide formation, which is consistent with previous observations.^19^ Specifically, our results identified >300 β-casein-derived peptides for all 10 samples, with several samples containing >500 β-casein-derived peptides. Similarly, α_s1_- and α_s2_-casein-derived peptide sequences were identified in all samples but with lower frequency (about 400 and 200 peptides per sample, respectively) (Fig. 4a). Minor proteins like glycosylation-dependent cell adhesion molecule-1 (GLCM1), polymeric immunoglobulin receptor (PIGR), butyrophilin (butyrophilin sub-family 1, member A1), osteopontin, perilipin-2 and serum amyloid-A also contributed to the total peptide count (Fig. 4b). Supplementary Table s2 shows that other milk proteins like α-lactalbumin, lactoferrin and mucin 1 were more resistant to proteolysis, probably due to their globular structure, yielding only few peptides overall (respectively: 1 peptide from α-lactalbumin in *Chunzhen (Yoghurt)*; 1 peptide from lactoferrin in *Huanqing for bone;* and 1 peptide each from mucin 1 in *Deluxe Organic Milk, Pure Milk*, *Xin Yang Dao, Chunzhen (Yoghurt) and Deluxe Milk*).

### Identification of bioactive peptides: homology to functional sequences

**Fig. 5:**
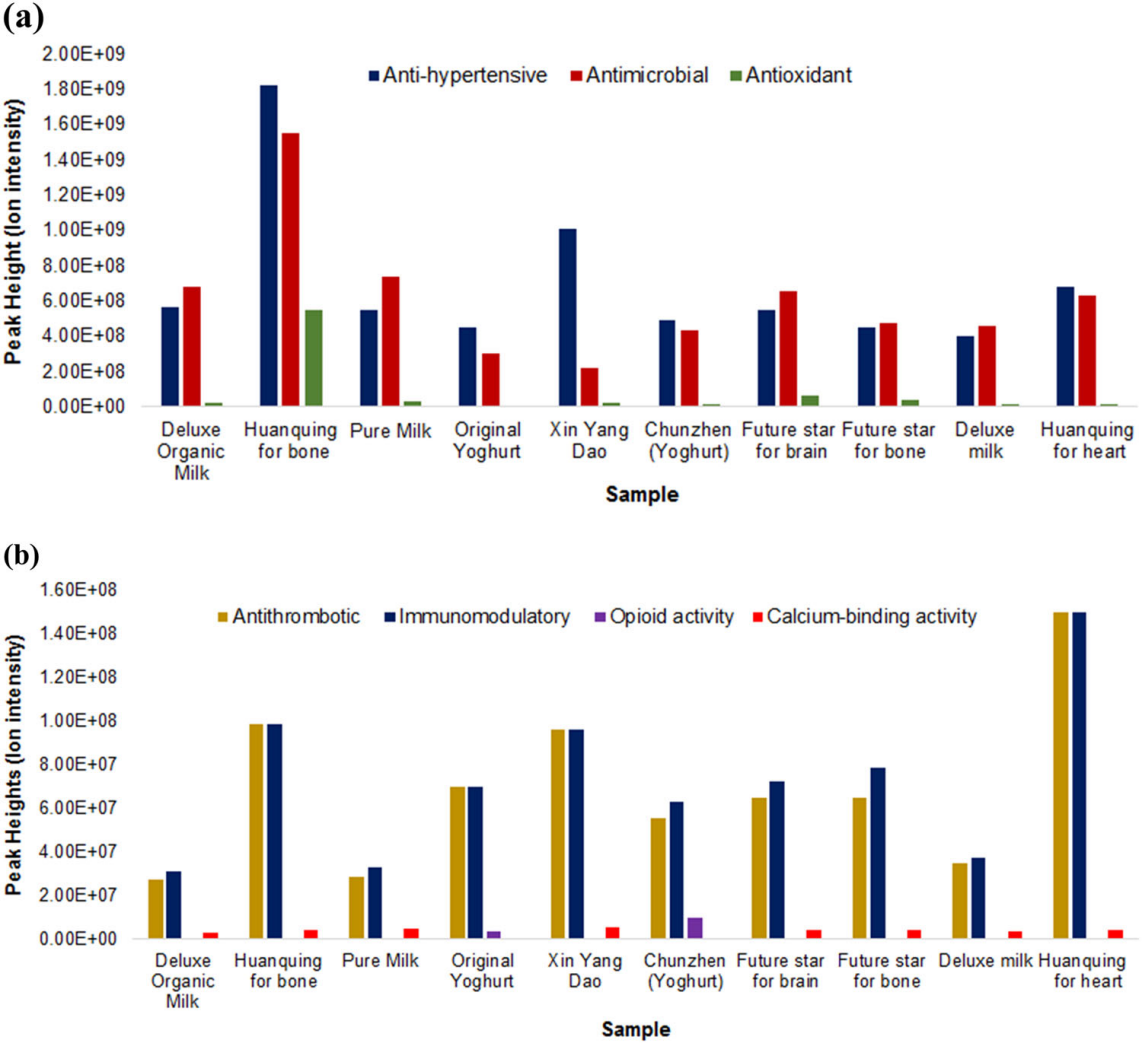
Relative abundances of functional peptides identified by LC–Orbitrap MS/MS in the 10 commercial dairy products. Peptides contributing to **a** anti-hypertensive, anti-microbial and anti-oxidant, **b** anti-thrombotic, immunomodulatory, opioid and calcium-binding activity were identified in 10 commercial dairy products analyzed

Sixty-six functional (bioactive) sequences were identified in the samples, including reported activities such as anti-hypertensive, anti-microbial, immunomodulatory, anti-thrombotic, anti-oxidative and opioid agonist functionalities (as presented in Supplementary Table s3). Table 2 provides the number of functional milk peptides identified in each sample. Figures 5a, b show the total relative abundances (peak height) of the peptides from each functional category.

**Table 2:** Number of functional milk peptides identified by LC–Orbitrap MS/MS in each commercial dairy products, grouped by function

Product name	Anti-hypertensive	Anti-microbial	Anti-oxidant	Anti-thrombotic	Immunomodulatory	Opioid agonist	Calcium-binding activity
Deluxe Organic Milk	20	15	2	1	2	0	1
Huanqing for bone	28	20	4	1	1	0	1
Pure Milk	24	17	2	1	2	0	1
Original Yoghurt	14	13	0	1	1	1	0
Xin Yang Dao	25	15	3	1	1	0	1
Chunzhen (Yoghurt)	18	15	3	1	1	1	0
Future Star for brain	25	15	2	1	2	0	1
Future Star for bone	23	15	1	1	2	0	1
Deluxe Milk	21	14	1	1	2	0	1
Huanqing for heart	25	16	2	1	1	0	1

Peptide sequences identified in the samples were matched against an in-house milk bioactive peptide database search program, which compares the identified peptides with sequences that are known to be bioactive

### Glycomic profiling

Table 3 presents the composition of OSs identified in all 10 commercial dairy commercial dairy products, as well as their relative abundance.

**Table 3:** Relative abundance of oligosaccharide classes (neutral, acidic and fucosylated) in all the samples analyzed by nano LC Chip Quadrupole - Time of Flight (QToF)

	Deluxe Organic milk	Huanqing for bone	Pure Milk	Original Yoghurt	Xin Yang Dao	Chunzhen (Yoghurt)	Future Star for brain	Future Star for bone	Deluxe Milk	Huanqing for heart
Oligosaccharides relative abundance (%)										
Neutral	70.50 ± 7.122	73.56 ± 5.387	70.32 ± 5.479	91.36 ± 8.592	83.88 ± 8.073	77.29 ± 4.901	79.35 ± 9.326	80.35 ± 8.591	66.00 ± 5.845	72.22 ± 5.058
Acidic	29.31 ± 3.428	26.27 ± 1.548	29.52 ± 3.774	8.63 ± 1.388	11.54 ± 1.256	22.63 ± 1.844	20.65 ± 2.995	19.65 ± 2.007	33.73 ± 3.011	26.44 ± 1.094
Fucosylated	0.22 ± 0.035	0.17 ± 0.0216	0.16 ± 0.0138	0.0214 ± 0.006	4.58 ± 0.387	0.08 ± 0.006	n.d.	n.d.	0.27 ± 0.032	1.36 ± 0.0844

Abundances are considered as the sum of all Oligosaccharide (including isomers) for each class Results expressed as mean ± standard deviation (*n* = 3)

*n.d.* not detected

### OSs profiling by nano LC Chip Quadrupole - Time of Flight (QTOF) MS

Sixty-nine unique OS structures, including isomers and anomers corresponding to 24 unique OS compositions were identified in all the samples analyzed. This information is made available in Supplementary Table s4. The samples *Pure Milk, Original Yoghurt, Deluxe Milk* and *Huanqing for heart* showed the highest OS diversity with 22 unique OS compositions, followed by *Deluxe Organic Milk (21), Chunzhen (21), Future Star for brain (16), Future Star for bone (16)* and *Huanqing for bone (15)*. Interestingly, the only sample that had been processed to be lactose free: *Xin Yang Dao* displayed the lowest OS diversity (13). Table 3 presents the relative abundance of OS classes (split in neutral, acidic and fucosylated) in all the samples as analyzed by nano LC Chip QTOF. Neutral OSs were the most abundant in all the samples (66–91.3%), followed by acidic (8.6–33.7%), and fucosylated OS (0–4.6%) (Table 3).

**Fig. 6:**
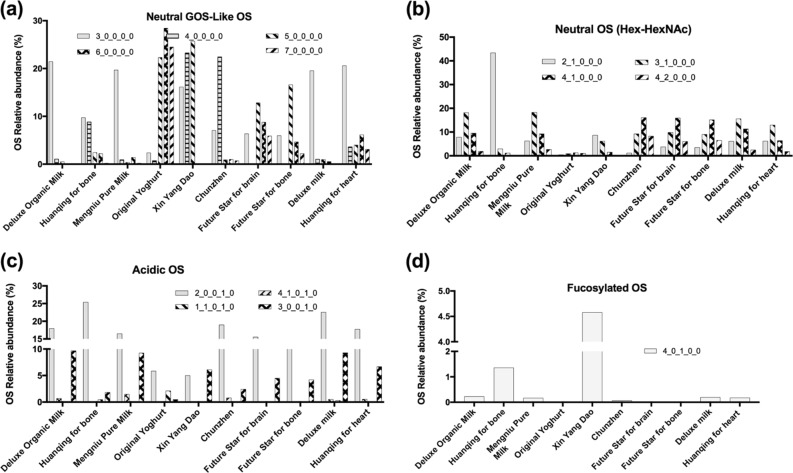
Relative abundance (%) of the various classes of oligosaccharides measured by nano LC Chip-QToF in the 10 commercial dairy products. **a** Neutral GOS-like OS, **b** acidic OS, **c** neutral OS with Hex and HexNAc and **d** fucosylated OS

Figure 6 presents a more detailed picture of OS distribution by further subdividing the neutral compounds into the categories of GOS-like (galacto-OSs, which only contains hexoses like glucose and galactose, Fig. 6a), and neutral HexNAc (*N*-acetylhexosamine)-containing OS (Fig. 6b). Figure 6a shows that similar relative abundance of hexoses with lower degree of polymerization (three hexose monomer) was noticed in most of the milk-based samples (*Deluxe Organic Milk*, *Pure Milk*, *Xin Yang Dao*, *Deluxe Milk* and *Huanqing for heart*), ranging from a low of 16.1% in lactose-free sample, *Xin Yang Dao* to 21.4% in *Deluxe Organic Milk*. Whereas, hexoses with higher degree of polymerization (4–10 hexose monomers) were mostly abundant in yogurts (*Original Yoghurt* and *Chunzhen*) and in one of the milk-based samples, lactose-free *Xin Yang Dao*. However, high relative abundance of four hexose monomer was observed in only one of the yogurts, *Chunzhen* (22.4%), as well as in lactose-free sample, *Xin Yang Dao* (23.2%). Similarly, only five hexose was noticed in lactose-free sample, *Xin Yang Dao* (25.6%). Whereas, high relative abundance of five, six and seven hexose monomers were observed in *Original Yoghurt* with values of 22.3, 28.4 and 24.4%, respectively. Figure 6b shows four neutral OS containing HexNAc in their structure, among all the 10 samples, *Huanqing for bone* was noticed to have exceptionally high relative abundance of acetylgalactosaminyl-α-1,3-galactose-β-1,4-glucose (2Hex–1HexNAc; 43.4%). Neutral OS with composition 3_1_0_0_0 and 4_1_0_0_0 were identified in all the samples with abundances ranging from 0.8 to 18.3% and 1.1 to 16%, respectively. Whereas, *Chunzhen (Yoghurt)* and pasture milk samples (where pasture-based diet were fed to cows), *Future Star for brain* and *Future Star for bone* were noticed to have higher relative abundance of 4_2_0_0_0 with values of 8.2, 6 and 6.4%, respectively. Figure 6c describes the four acidic OS as identified by nano LC Chip QTOF. Among these four identified acidic OS structures, sialyllactose (SL, composition 2Hex 1 sialic acid) was the most abundant overall when considering individual OS found in all the 10 samples, with abundances comprising 5–25.3% of the total OS content. Acidic OS with composition 3_0_0_1_0 was noticed in all the samples, high relative abundances were observed for *Deluxe Organic Milk* (9.6%), *Pure Milk* (9.2%) and *Deluxe Milk* (9.2%). Whereas, acidic OS with composition 4_1_0_1_0 and 1_1_0_1_0 were found in trace levels in most of the commercial dairy products. Similarly, single acidic OS with NeuGc (*N*-glycolylneuraminic acid) in its composition (3_0_0_0_1) was found in trace levels (0.07–1.45% of total OSs) in *Deluxe Organic Milk, Pure Milk*, *Original Yoghurt*, *Xin Yang Dao*, *Deluxe Milk* and *Huanqing for heart* (as presented in Supplementary Table s4). The single fucosylated OS structure (4_0_1_0_0) identified in the present set of samples is presented in Fig. 6d (and also Supplementary Table s4) demonstrates that the lactose-free sample, *Xin Yang Dao* have a unique OS composition compared with the rest of the samples evaluated. The unusually high abundance of fucosylated OS observed in the zero-lactose sample (4.6%) represents the highest amount reported in a bovine milk product.^14^

### OS quantification by HPAEC-PAD

Six neutral (GalNAc(α1-3)lactose, galactose-α-1,3-galactose-β-1,4-glucose (3Hex), Lacto-*N*-neotetraose (LNnT), Lacto-*N*-tetraose (LNT), Lacto-*N*-neohexaose (LNnH), Lacto-n-Hexaose (LNH) and four acidic OSs (6ʹ-sialyllactosamine (6ʹ-SLN), 3ʹ-sialyllactosamine (3ʹ-SLN), 6ʹ-sialyllactose (6ʹ-SL), 3ʹ-sialyllactose (3ʹ-SL)) that are known to exist in bovine milk and for which commercial standards are available, were measured by high-performance anion-exchange chromatography coupled to pulsed amperometric detector (HPAEC-PAD). Samples displayed a strong variability in both individual and total OS content (Table 4). 3ʹ-SL was the only OS quantified in all the samples analyzed, whereas LNH was not detected in any sample. 2Hex–1HexNAc and 6ʹ-SL was quantified in 90% of samples, 3Hex in 70%, 6ʹ-SLN, LNT and LNnT in 60% and 3ʹ-SLN in 40% of the samples. Neutral OS LNnH was not detected in any sample, expect for *Huanqing for heart*, and the amount quantified was 5.6 mg/L. OS with composition 2Hex–1HexNAc was found to be highest in the zero-lactose *Xin Yang Dao* and *Pure Milk* samples (14.1–16.6 mg/L). The apparent contradiction with the results obtained by QTOF, where *Huanqing for bone* was observed as having the highest abundance of 2Hex–1HexNAc is explained by the presence of 2–5 identified isomers (refer to Supplementary Table s4); all measured by QTOF, yet only one of those is commercially available as pure standard that can be used for absolute quantification. The total OS concentration varied among samples, ranging from 24.8 to 80.3 mg/L. Remarkably low OS concentration were observed in the yogurt samples (24.8 and 37.3 mg/L for *Chunzhen* and *Original Yoghurt*, respectively). The sample with the highest OS concentration was *Future Star for brain* (80.3 mg/L).

## Discussion

Milk-derived peptides provide an attractive target given their recognized ability to provide anti-hypertensive, anti-inflammatory, anti-diabetic, anti-oxidant properties, opioid activities,^20–22^ as well as their “built in” safety dossier. In the present study, we identified a multitude of peptides in 10 commercial dairy products by employing high-resolution Orbitrap mass spectrometry and related peptidomic techniques (Table 1). We have reported the highest number of peptides for commercial dairy samples to date, with an average of 1732 peptides, which represents the most comprehensive peptidomic profiling of milk in the current literature. Only peptidomic studies examining fermented dairy products have uncovered similar peptide profiles to those in the present study: a study on the peptide profile of the yogurt Feng Wei Suan Ru obtained from a Chinese dairy identified 250 peptides in yogurt by using a linear ion trap–Orbitrap XL mass spectrometer.^23^ Our group has previously investigated the proteolytic activity of kefir microorganisms, and shown an average peptide count of 1421 ± 42 for kefir samples by applying similar analytical techniques to those used in this study.^24^ These findings illustrate the capability of modern high-resolution MS platforms to provide in-depth analysis of proteomic and peptidomic samples without the need for prior fractionation or multiple LC-MS runs for each sample.

As previously discussed in the Results section, most of the peptides identified using mass spectrometry originated from caseins, and a few from whey proteins. Cation-exchange chromatography enabled separation and quantification of amino acids from acid-hydrolyzed peptides in individual samples. The amounts of peptides, as represented by amino acids derived from 10 commercial dairy products ranged from 60 to 130 mg/L as measured by ion-exchange chromatography (Fig. 3). However, along with the amino acids derived from peptides, it may contain free amino acids. Figures 1 and 3 summarize the results for peptides characterization and quantification, respectively. *Huanqing for bone* had highest peptide count (2117 peptides), as well as highest concentration of peptides (130 mg/L). In addition to naturally occurring peptides, casein phosphopeptides as an ingredient of *Huanqing for bone* likely contributed to the peptide count. However, the peptide counts of *Pure Milk* (1695 peptides) and *Xin Yang Dao* (1769 peptides) were different though both had lowest peptide content (60 mg/L). This suggests that although *Xin Yang Dao* contributed a higher number of peptide sequences, the concentration of individual sequences was low as compared with *Pure Milk*. These results corroborated well with the relative quantification of peptides in the 10 dairy products.

Milk proteins have diverse structures and their conformation can directly affect their propensity to undergo proteolysis. The simple rheomorphic structure of caseins make them more susceptible to proteolysis, unlike the tightly packed complex globular structures of α-lactalbumin, β-lactoglobulin, and other whey proteins. The post-translational modifications (glycosylation in particular) of lactoferrin further influence its resistance to protease activity.^25^ These structural differences are reflected in the fact that casein-derived peptide sequences were most frequently identified in the present sample set. The peptides identified in these 10 commercial dairy samples were derived from 63 milk proteins. The proteins α_s1_-casein, α_s2_-casein, β-casein, κ-casein, β-lactoglobulin, GLCM1, PIGR, butyrophilin, osteopontin, perilipin-2, serum amyloid-A and lactoperoxidase were the major sources of peptides (Supplementary Table s2). The signal peptide sequences of α_s1_-, α_s2_- and β-casein are amino acids 1–15, whereas the signal sequence for κ-casein is amino acids 1–21. The signal peptide sequence is an additional peptide sequence at the amino terminus of the precursor protein that facilitates direct translocation of generated proteins across the cytoplasmic membrane.^26^ In this article, protein signal sequences are included in the amino-acid numbering of all peptides.

Among whey proteins, β-lactoglobulin has a complex three-dimensional structure that consists of a barrel shaped core made up of eight anti-parallel β-sheets with a short strand of α-helix on the surface. Most of the previous studies on bovine milk did not identify any peptides derived from β-lactoglobulin.^19,27–29^ However, this study identified the highest number of peptides to date (29 peptides) from β-lactoglobulin in the product *Xin Yang Dao*. Similarly, previous studies on bovine milk did not identify any peptides from lactoferrin or mucin 1.^19,28,29^ However, peptides from lactoferrin and mucin 1 were reported in the present study, as previously discussed in the Results section. The number of peptides identified from the most highly represented proteins is shown in Figs. 4a, b. The number of proteins from which the identified peptides originated was also higher as compared with previous studies, and this can be attributed to the enhanced high-resolution afforded by LC–Orbitrap MS/MS analysis and targeted data analysis by searching the tandem spectra against an exhaustive library of bovine milk proteins with X!Tandem.^19,29,30^

Sixty-six functional sequences were identified using a milk bioactive peptide database in the 10 commercial dairy products. A few of the peptide sequences were identified to have multiple bioactive functions. These bioactive peptides may be released by endogenous enzymes mainly plasmin, cathepsin B, cathepsin D, or elastase present in bovine milk.^19^ In yogurts, mainly bacterial fermentation is responsible for modeling peptidomic profile. Proteolytic activity of cell wall-bound proteinases and intracellular peptidases of lactic acid bacteria, such as *Lactococcus lactis, Lactobacillus delbrueckii* var. *bulgaricus* and *Lactobacillus helveticus*, is well established.^31,32^ Identified bioactive peptides and their functionalities are discussed below:

Anti-hypertensive peptides are the most studied among bioactive peptides. These peptides can inhibit the angiotensin I-converting enzyme (ACE), a multifunctional ectoenzyme that catalyzes the conversion of angiotensin I to angiotensin II. Production of the angiotensin II results in vasoconstriction; in addition, ACE also reduces the vasodilatory properties of bradykinin, which collectively increases the blood pressure.^33^ Substrates or competitive inhibitors like tripeptides with hydrophobic amino acids such as Valine and Isoleucine at the amino terminus were found to inhibit ACE activity.^34^ However, more studies elucidating the structure–activity relationship of ACE-inhibitory peptides have yet to be conducted. To date, many peptides derived from bovine and human caseins have been identified to be ACE inhibitors.^35^

In the present study, the anti-hypertensive peptide β-casein (184–190) was identified in most samples, except for *Deluxe Organic Milk, Pure Milk, Original Yoghurt* and *Deluxe Milk*. A previous research reported anti-hypertensive activity for β-casein (184–190), with an IC_50_ value of 1000 μM, after oral administration of 2 mg peptide/kg of body weight in spontaneously hypertensive rats.^36^ Similarly, β-casein (123–128) was found to exhibit bradykinin-potentiating activity^18^ and was identified in all the samples except for the two yogurt samples, *Original Yoghurt* and *Chunzhen*. This bioactive sequence was observed to be a competitive inhibitor of ACE and putative bradykinin potentiator.^18,19^ A previous study analyzed the ACE-inhibitory activity of β-casein (208–224), also known as casecidin-17, and found that it exhibited an inhibition efficiency of 0.14%/peptide concentration (μg/mL).^37^ Casecidin-17 was found in highest abundance (1.5E + 08 ion intensity) in *Huanqing for heart*. Most of the anti-hypertensive peptide sequences in *Huanqing for bone* and *Huanqing for heart* came from β-casein. The anti-hypertensive peptides identified in *Huanqing for bone, Xing Yang Dao* and *Huanqing for heart*, (28, 25 and 25, respectively), contributed to the highest relative abundance as compared with rest of the samples (Fig. 5a). These studies have not been conducted on humans, so further studies are needed to determine their effectiveness.

Many researchers have described the beneficial activity of anti-microbial peptides in improving the health of the host animal by affecting the gut microflora. Their selective activity suppresses the growth of harmful Gram-positive bacteria like *Clostridium* spp. and *Staphylococcus* spp., Gram-negative bacteria like coliforms, fungi and mycobacteria while favoring the growth of beneficial commensal-gut microbiota like *Lactobacillus* and *Bifidobacterium.*^38–43^ The mechanism of action against Gram-negative bacteria may be due to the interaction between the positively charged side chains of lysine, arginine and histidine on C-terminal of peptides and the negatively charged bacterial cell wall or membranes, mostly composed of lipopolysaccharides.^44–46^

The peptide α_s2_-casein (196–222), also known as Cr1, has anti-microbial activity^47^ and was found only in *Chunzhen (Yoghurt)*, which suggests that break down of milk proteins during fermentation by lactic acid bacteria might be responsible for its generation. β-Casein (192–198) and β-casein (206–220), also known as β-casokinin-7 and casecidin-15, respectively, were found in highest abundance (1.27E + 08 and 3.06E + 07 ion intensity, respectively) in *Huanqing for bone*. Similarly, β-casein (208–224), also known as casecidin-17 was present in highest abundance in *Huanqing for heart* (1.5E + 08 ion intensity). A study showed casecidin-15 and casecidin-17 to be effective against *E. coli.*^48^

The 20 anti-microbial peptides identified in *Huanqing for bone* contributed to the highest relative abundance as compared with rest of the samples (Supplementary Table s3). If proven effective in vivo, one could possibly envision future application of these anti-microbial peptides as a potential substitute of antibiotic drugs, since the increased use of antibiotics to treat infection over the last few decades had led to subsequent antibiotic resistant bacteria, posing a threat to human health. Human deaths due to antibiotic-resistant infections are predicted to reach 10 million by year 2050, which is reported to be higher than the deaths caused due to cancer.^49^ Thus, anti-microbial peptides could be a possible answer to solving antibiotic resistant infections.

Another set of notable functional peptides are anti-oxidative, mostly derived from β-casein, such as β-casein (192–198), β-casein (184–191) and β-casein (113–120),^50^ which were also reported in the present study. The four anti-oxidative peptides identified in *Huanqing for bone* contributed to the highest relative abundance as compared with rest of the samples (Supplementary Table s3). However, the literature on in vivo effects of anti-oxidant peptides is still limited.

Several studies have investigated the similarities between clotting of blood and milk, suggesting functional homologies between peptide fragments of fibrinogen and κ-casein.^51,52^ For example, the κ-casein-derived peptide casoplatelin (not found in this study) was shown to inhibit fibrinogen binding to platelets.^52^

All the 10 commercial dairy products contained β-casein (208–224), which has anti-thrombotic activity.^37^ Further in vivo studies need to be conducted to prove the efficacy of β-casein (208–224) in human subjects.

A study showed α_s1_ casein, β-casein and α-lactalbumin-derived peptides protect against *Klebsiella pneumoniae* infection in mice by influencing phagocytic activities of murine and human macrophages.^51,53^ β-Casein (208–224), also known as casecidin-17 is an immunomodulatory peptide^19^ and was present in the highest abundance in *Huanqing for heart* (1.5E + 08 ion intensity). In a previous study, β-lactoglobulin (100–107) was observed to have proliferative effect on murine splenocytes.^54^ It was present in *Deluxe Organic Milk, Pure Milk, Future Star for brain, Future Star for bone* and *Deluxe Milk*.

A study demonstrated that a few casein-derived peptides had opium (morphine) and naloxoneinhibitable properties.^7,53^ The opioid agonists are mainly α- and β-casein-derived peptides, mostly casomorphins, whereas opioid antagonists are known as casoxins.^55,56^ Hydrolysis of casomorphins in gut leads to the generation of smaller bioactive fragments. These fragments then react with µ- and δ-type receptors located in the gut and in the brain,^7^ which may further affect social behavior, and have physiological effects including analgesia, increased secretion of insulin and somatostatin influencing postprandial metabolism.^56^

A previous study reported the presence of the opioid peptide β-casein (109–138), a precursor of the opioid agonist neocasomorphin-6, in a marketed yogurt.^54^ Similarly, we revealed the presence of β-casein (109–138) in *Original Yoghurt* and *Chunzhen (Yoghurt)*, confirming that the peptide is resistant to complete fermentation by lactic acid bacteria; however, it cannot be excluded that smaller fragments could be released during human digestion.

The negatively charged side chains of phosphopeptides bind to positively charged minerals. These phosphopeptides form soluble complexes with calcium, which prevents its proteolysis in the gut, thus, enhancing calcium bioavailability through intestinal absorption.^56^ The casein phosphopeptides were shown to mitigate dental caries and oral diseases by preventing biofilm formation.^57^

In β-casein (16–40), a casein phosphopeptide was present in all the samples except for *Original Yoghurt* and *Chunzhen (Yoghurt)*, the first four negatively charged phosphorylated serine residues help to form peptide–calcium complex by binding to the positively charged calcium, facilitating calcium transport throughout the body.^58,59^ Casein phosphopeptides and vitamin D being important ingredient of *Huanqing for bone* may result in strengthening bones in the elderly population (Table 1).

Despite being minor components of bovine milk, OSs have attracted much attention due to the biological processes they are involved in, especially in the first stages of life.^3^ It has been proved that milk OSs have an active role in the gastrointestinal tract exhibiting prebiotic, anti-adhesive, immunomodulatory and cognitive development activity in humans.^2,4^ Due to their complex structure (monomer composition and linkages), their identification and quantification have been proved challenging. Recent technological developments placed mass spectrometry as one of the most valuable and widely used tools for the characterization of OS in mammalian milk.^12,14,60^

The nano LC Chip QTOF MS system has been shown to be an excellent tool for OS characterization in many mammal milks, allowing for the identification of as many as 150 different OS in human milk.^61,62^ Raw bovine colostrum is known to contain predominantly acidic OS (50–70%), with a lower abundance of neutral (25–40%) and fucosylated OS (found at the trace level: <1%).^14^ Given that formulations and processing can vary significantly in commercial dairy products, it can be expected that there could be a variation in OS composition and abundances, but the OS profile is expected to resemble that of raw bovine milk. Thus, further establishing the fact that OSs are relatively heat stable and match closely to the OS profile of raw bovine milk, even after being subjected to thermal treatment during industrial processing. However, there is no available literature on heat stability of bovine milk OSs but previous publications on thermal treatment of human milk OSs and glycolipids report that both are thermally stable.^63,64^ The commercial dairy products analyzed (Table 3), obtained from mature bovine milk, displayed a higher abundance of neutral (66–91.3%) OS and lower abundance of acidic ones (8.6–33.7%), with fucosylated present only at the trace level, which is in agreement with previously published work on Holstein mature milk.^65^

As mentioned in the Results section, neutral OS GOS-like structures with lower (3 hexoses) and higher degree of polymerization (4–10 hexoses) were found in most of the milk-based (*Deluxe Organic Milk*, *Pure Milk*, *Xin Yang Dao*, *Deluxe Milk* and *Huanqing for heart*) and both yogurt samples (*Original Yoghurt* and *Chunzhen*), as well as lactose-free sample, *Xin Yang Dao*, respectively (Fig. 6a). Thermal processing followed by an additional step such as fermentation by bacterial enzymes or lactose removal using enzymes like β-galactosidase employed in the production of yogurt samples (*Original Yoghurt* and *Chunzhen*) and lactose-free sample, *Xin Yang Dao*, respectively, could probably be related to the formation of neutral GOS-like structures with higher degree of polymerization (4–10 hexoses). A reported study on enzymatic activity of beta-galactosidase and their ability to form GOS from lactose supports our finding,^66^ as they are simpler in structure and thus, a broader array of bacteria compared with bovine and human milk OSs could utilize them. However, when paired with an appropriate probiotic strains, it may work as a selective synbiotic.^67^ Identification of these specific enzymes responsible for OS remodeling, could enable us to increase GOS amounts during commercial production. The high relative abundance (0–43.4%) of OS containing the so-called Bifidus factor (HexNAc), for which specific in vitro prebiotic activity has been demonstrated,^68^ is remarkable (Fig. 6b), especially in the case of the OS with structure 3Hex–1HexNAc (0.8–18.1%), implicating selective consumption by OS-consuming bifidobacterial strains. This relationship, if better understood, could be exploited for commercial production. Figure 6c show *Huanqing for bone*, displayed a highest content of acidic OS, 3ʹ-SL. A previous publication on functional role of siallylactose summarizes effect of siallylactose and other sialylated OS on brain and cognitive development.^69^ Single acidic OS with NeuGc in its OS composition was quantified in trace levels in all the samples, a similar finding was reported for a study on variation in OS level during different stages on lactation in mature bovine milk.^70^ *Xin Yang Dao* had highest relative abundance of fucosylated OS (4.6%) as shown in Fig. 6d. The factors leading to the unusually elevated proportion of fucosylated OS in the *Xin Yang Dao* sample are unclear but could probably be related to the unspecific activity of enzyme employed in the lactose removal process. Human milk fucosylated OS, 2ʹ Fucosyllactose in a clinical trial showed that infants fed on Similac infant formula supplemented with a structurally identical version of human milk OS 2ʹ-FL, had immunomodulatory effects similar to breast-fed infants.^71^

These data also suggest that *Xin Yang Dao* may be good starting product to use for the creation of synbiotic products containing OS-consuming bifidobacterial strains. Further optimization of the enzyme would allow manipulating the lactose hydrolysis reaction to increase production of GOS, as well as more bioactive fucosylated OSs. The variation in OS composition and abundances observed in the *Xin Yang Dao* as compared with the other dairy samples in the set, are possibly due the effect of various ingredients used in the formulation and processes they were subjected to (e.g., enzymatic hydrolysis of lactose for *Xin Yang Dao*).

In order to minimize OS loss during industrial processing and ideally reach a pattern closer to human milk, more methodological studies need to be carried out to elucidate the precise factors behind the OS remodeling/increase or decrease.

Complementary information to OS profile can be obtained by HPAEC-PAD, which has become also a popular tool, thanks to its sensitivity and specificity for carbohydrates.^72,73^ Unfortunately, accurate quantification requires identical pure standards and the synthesis of these class of compounds has been demonstrated difficult due to their complex structure and stereospecific chemistry, with only a handful of OS with the required purity being commercially available. The total amount of OS measured in this sample set was consistent with the OS content described for mature bovine milk,^66,72,74^ although some differences were found among samples (Table 4). The lower OS content in the yogurt (*Chunzhen*) (24.8 mg/L) and *Original Yoghurt* (37.27 mg/L) might be attributed to the fact that bacteria in yogurt may consume OS.

**Table 4:** Concentration of key oligosaccharides in the dairy samples analyzed by HPAEC PAD

Samples	2Hex–1HexNAc	3Hex	LNnT	LNnH	LNT	6′-SLN	3′-SLN	6′-SL	3′-SL	Total OS
Oligosaccharide concentration (mg/L)										
Deluxe Organic milk	3.45 ± 0.031	5.01 ± 0.04	16.67 ± 0.57	n.d.	n.d.	1.35 ± 0.02	0.71 ± 0.11	6.91 ± 0.25	22.4 ± 0.69	56.5 ± 1.2
Huanqing for bone	3.63 ± 0.25	1.77 ± 0.61	n.d.	n.d.	n.d.	0.97 ± 0.21	9.27 ± 0.65	8.27 ± 0.67	22.40 ± 1.35	46.31 ± 2.55
Pure Milk	14.1 ± 0.99	3.1 ± 0.85	n.d.	n.d.	5.3 ± 0.71	n.d.	n.d.	11.4 ± 1.27	21.6 ± 1.84	56.55 ± 5.73
Original Yoghurt	9.7 ± 0.95	8.1 ± 0.56	n.d.	n.d.	5.47 ± 0.83	1.17 ± 0.21	n.d.	3.47 ± 0.7	9.37 ± 1.91	37.27 ± 3.17
Xin Yang Dao	16.56 ± 0.87	6.89 ± 0.48	9.85 ± 0.27	n.d.	1.5 ± 0.09	n.d.	n.d.	5.8 ± 0.31	25.18 ± 0.94	65.78 ± 2.44
Chunzhen (Yoghurt)	1.20 ± 0.01	0.83 ± 0.06	n.d.	n.d.	n.d.	2.43 ± 0.12	3.97 ± 0.15	4.57 ± 0.12	11.8 ± 0.70	24.82 ± 0.95
Future Star for brain	3.4 ± 0.62	n.d.	5.93 ± 0.59	n.d.	28.17 ± 1.07	n.d.	n.d.	6.13 ± 0.65	36.63 ± 1.87	80.27 ± 4.03
Future Star for bone	3.5 ± 0.87	n.d.	6.7 ± 0.44	n.d.	25.17 ± 2.29	n.d.	n.d.	5.2 ± 0.26	37.53 ± 4.7	78.1 ± 6.29
Deluxe Milk	3.91 ± 0.23	5.60 ± 0.29	17.5 ± 0.26	n.d.	n.d.	1.69 ± 0.11	0.81 ± 0.10	8.84 ± 0.55	26.8 ± 1.47	65.15 ± 3.31
Huanqing for heart	4.8 ± 0.35	6.93 ± 0.42	n.d.	5.6 ± 0.1	n.d.	1.53 ± 0.23	n.d.	5.0 ± 0.35	20.0 ± 1.06	44.33 ± 2.08

Results expressed as mean ± standard deviation (*n* = 3)

*n.d.* not detected

Overall, the dataset appeared to have a rather diverse distribution in terms of individual OS prevalence in the samples: while 3ʹ-SL, 6ʹ-SL and 2Hex–1HexNAc were present in most of the samples, LNH was not at all quantifiable whereas other OS for which a commercial standard exist, were scattered in terms of concentration. Interestingly, a unique feature of pasture milk samples was the unusual high abundance of LNT (~26 mg/L in *Future Star for brain* and *Future Star for bone)*. These higher values might be the result of the different composition of cows’ diet. A recent study investigating the composition of milks produced from pasture-fed cows in either organic or conventional production systems concluded there was variability among farm system and it appeared to have an effect on some OS, although the differences were not significant enough to make a strong statement.^75^

When comparing the overall OS profile with the absolute quantification, one must remember that the paucity of commercial standards impedes the quantification of several key compounds identified by nano LC Chip QTOF MS. Considering that no commercial standards for GOS-like OSs are available, 3ʹ-SL was measured as the most abundant OS, followed by 2Hex–1HexNAc and 3Hex–1HexNAc–LNT and isomers.

In conclusion, the commercial dairy products tested are potentially a source of prebiotic OSs, as well as functional peptides, with a variety of attributed functions such as anti-hypertensive, anti-bacterial, calcium-binding, opioid agonist, anti-oxidant and immunomodulatory. However, the functional activity of these peptides that was observed in vitro may be degraded during the process of digestion and absorption in the body.^76^ With this information in hand, further studies need to be carried out to assess whether the concentration of OSs and peptides naturally found in dairy products is sufficient to provide a natural solution to hypertension, gastrointestinal infections and other related disorders. Peptides and OS in commercial dairy products vary possibly due to different processes and formulations, products with similar formulation and process show similar content and profile as bovine milk. If better understood, processing conditions and the use of enzymes and bacterial cultures on the peptide formation and OS remodeling would reveal opportunities for tailored processes to increase peptide and OSs content and develop enriched food products with health-promoting activities targeted for specific consumer populations.

## Methods

Ten commercial dairy products were provided by Mengniu Dairy Company Limited, Hong Kong, China. These dairy products were derived from milk of Holstein cows. The main characteristics of these commercial dairy samples are reported in Table 1.

### Peptidomic profiling

#### Samples

Ten commercial dairy products comprised of eight milk-based and two yogurt samples were used for peptidomic profiling and quantification.

#### Sample preparation

Peptides were extracted from the dairy products essentially as described previously by Dallas et al.^77^ with the following exceptions: 100 µL of skimmed sample was combined with 400 µL water and spiked with 4 µL of 10 µg/mL peptide internal standard (Proteochem, Loves Park, IL, USA). However, the peak height (ion intensity) of identified peptide in the samples were not normalized to internal standard, since peak height (ion intensity) of internal standard was considerably higher than the average peak height (ion intensity) of identified peptides in the samples. To this mixture, four volumes (2.016 mL) of a 2:1 chloroform:methanol mixture were added. Prior to protein precipitation with trichloroacetic acid, samples were redissolved in 400 μL nanopure water. An equal volume of 200 g/L trichloroacetic acid was added, and the samples were centrifuged at 4000 × *g* and 4 °C for 30 min. The supernatant, containing the naturally occurring peptides, was transferred to a new tube, and 0.6 mL of each supernatant was purified by microplate C18 (Glygen^TM^ Corp., Columbia, MD, USA) solid phase extraction (SPE) as described previously by Dallas et al.^77^ Salts, sugars and trichloroacetic acid were washed from the microplate with six column volumes of 1% acetonitrile (ACN)/0.1% trifluoroacetic acid (TFA). Peptide solutions were dried, and the samples were redissolved in 200 µL 2% ACN/0.1% TFA for relative and absolute quantification of peptides using LC–Orbitrap MS/MS and ion-exchange chromatography, respectively.

#### Peptide abundance determination by LC–Orbitrap MS/MS

The total peptide abundance in each sample was determined by a fluorometric peptide assay (Pierce™ Quantitative Fluorometric Peptide Assay, Eugene, OR, USA) and used to guide injection volumes were determined for each sample, such that 1 µg of peptides would be loaded for LC separation. Abundance of peptides were determined using mass spectrometry as described previously by Dallas et al.^24^

#### Spectral analysis and peptide identification

Spectral analysis and identification of peptides were performed as described previously by Dallas et al.^19^

#### Relative quantification of peptides

Skyline software was employed for the relative quantification of peptides. The peaks for peptides were extracted as described in a previous study with minor modifications.^24^ Peptides length was allowed to vary between 2 and 50 amino acids. The precursor charge was fixed from 1 to 7. The mass to charge ratio was allowed to vary between 50 and 1600 m/z with match tolerance of 0.055. The number of isotope peaks selected were 3, which included peaks for *M* (monoisotopic ion), *M* + 1, *M* + 2. Tandem MS filter was set to none. Skyline software was set with a retention time window of 5 min to select the peaks from tandem MS spectra.

After importing the data, a relative quantification was performed for peptides above a threshold of isotope distribution score (ratio between measured spectra and theoretical isotope patterns) ≥ 0.6 and peak height ≥ 2 × 10^6^ ion intensity. The peaks matching these criteria were included and were exported as.csv file. An in-house script was used to collapse multiple charge states for the peptides together and the peptide sequences were assigned based on the originating milk proteins.

#### Functional peptide annotation

Peptide sequences identified in the samples were matched against an in-house milk bioactive peptide database search program, which compares the identified peptides with sequences that are known to be bioactive.^78^ The peptides with a 100% match with the functional peptides were reported.

#### Absolute quantification of peptides by ion-exchange chromatography

A 50 µL aliquot of each sample after SPE were dried via speed vacuum concentration (SpeedVac, Genevac, NY, USA) and then acid-hydrolyzed using 6 N HCl (Pierce, Rockford, IL, USA), 1% phenol (Sigma-Aldrich, St. Louis, MO, USA) at 110 °C for 24 h.^79^ The acid-hydrolysates were vacuum-dried and then subsequently dissolved in sodium diluent (Pickering Laboratories, Mountain View, CA, USA) containing 40 nmol/mL norleucine (CalBioChem, La Jolla, CA, USA). Resultant amino acids were separated by ion-exchange chromatography (Hitachi 8800 Amino Acid Analyzer, Tokyo, Japan) with a postcolumn ninhydrin reaction for quantification. Amino-acid standards (Sigma-Aldrich, St. Louis, MO) in conjunction with the National Institute of Standards and Technology’s (NIST) standards were employed to calibrate the amino-acid analyzer. Individual amino acids in the samples were quantified against the known amino-acid standards; norleucine was used as a reference standard.

### Glycomic profiling

#### Chemicals and reagents

Sodium acetate and sodium hydroxide were from Fisher-Scientific (Fair Lawn, NJ, USA), ACN, chloroform and formic acid High Performance Liquid Chromatography (HPLC)-MS grade were purchased from Fisher-Scientific (Fair Lawn, NJ, USA). Electrospray Ionization (ESI)-TOF low concentration Tuning Mix G1969–85000 was purchased from Agilent Technologies (Santa Clara, CA, USA). Nanopure water (18.2 MΩ.cm, 25 °C) was used throughout all experiments.

Analytical grade standards ( > 99%) of LNnT, LNT, LNnH, 2Hex–1HexNAc, 3Hex, 6ʹ-SLN, 6ʹ-SL and 3ʹ-SL were purchased from V-Labs (Covington, LA, USA). Standard solutions and samples were filtered through nylon FH membranes (0.22 µm; Millipore, Bedford, MA, USA) before injection in the HPAEC-PAD system.

#### OS profiling by nano LC Chip QTOF MS and quantification by HPAE-PAD

Mengniu Dairy Company Limited, Hong Kong, China, provided 10 commercial dairy products. The dairy products comprised of eight milk-based products and two yogurt samples. Profiling and quantification of OS was performed as described previously by Mudd et al.^80^

### Reporting summary

Further information on research design is available in the Nature Research Reporting Summary linked to this article.

## Supplementary information

Supplementary Tables S1, S2, S3 and S4

Life Sci Reporting Summary

## Data Availability

The data sets generated during and/or analyzed during the current study are not publicly available but can be made available from the corresponding author on reasonable request.

## Supplementary information

**Supplementary information** accompanies the paper on the *npj Science of Food* website (10.1038/s41538-019-0037-9).
